# Dynamic tracking of a magnetic micro-roller using ultrasound phase analysis

**DOI:** 10.1038/s41598-021-02553-z

**Published:** 2021-12-01

**Authors:** Stefano Pane, Veronica Iacovacci, Mohammad Hasan Dad Ansari, Arianna Menciassi

**Affiliations:** 1 The BioRobotics Institute, Scuola Superiore Sant’Anna, 56025 Pisa, Italy; 2 Department of Excellence in Robotics and AI, Scuola Superiore Sant’Anna, 56025 Pisa, Italy; 3 Department of Mechanical and Automation Engineering, The Chinese University of Hong Kong, Shatin, Hong Kong SAR China; 4 Robot-Assisted Surgery Group, Department of Mechanical Engineering, KU Leuven, 3001 Leuven, Belgium

**Keywords:** Biomedical engineering, Acoustics

## Abstract

Microrobots (MRs) have attracted significant interest for their potentialities in diagnosis and non-invasive intervention in hard-to-reach body areas. Fine control of biomedical MRs requires real-time feedback on their position and configuration. Ultrasound (US) imaging stands as a mature and advantageous technology for MRs tracking, but it suffers from disturbances due to low contrast resolution. To overcome these limitations and make US imaging suitable for monitoring and tracking MRs, we propose a US contrast enhancement mechanism for MR visualization in echogenic backgrounds (e.g., tissue). Our technique exploits the specific acoustic phase modulation produced by the MR characteristic motions. By applying this principle, we performed real-time visualization and position tracking of a magnetic MR rolling on a lumen boundary, both in static flow and opposing flow conditions, with an average error of 0.25 body-lengths. Overall, the reported results unveil countless possibilities to exploit the proposed approach as a robust feedback strategy for monitoring and tracking biomedical MRs in-vivo.

## Introduction

Recent progress in microrobotics provided relevant medical perspectives for non-invasive therapy and diagnosis^1–3^. However, the translation of microrobotics technologies to the clinics is hampered by the lack of suitable medical imaging strategies to provide robust and precise feedback for monitoring and control purposes^4,5^. At present, most of the advancements made in the field of microrobotics have been enabled by direct optical feedback through microscopy^6–9^. Accomplishing a comparable resolution and image quality in an in-vivo setting by medical imaging techniques is a major challenge, still to be addressed. Several medical imaging techniques have been considered for this purpose^10^, ranging from traditional techniques (e.g., MRI^11^ or radiation-based^12^) to innovative ones, such as photoacoustic^13^ or magnetic particle imaging^14^. In this framework, medical ultrasound (US) stands as a mature technology that combines real-time capabilities with a good spatial resolution (100–500 μm), deep tissue imaging (up to $25\,\text{cm}$ far from the probe), no adverse health effects, and low equipment cost^15^, thus being a good candidate also for tracking microrobots (MRs) inside the body.

The gold standard for US imaging is Brightness (B)-mode, which has been employed to monitor MRs during navigation and cargo delivery tasks^16–22^. In B-mode, image contrast is associated with objects echogenicity, defined as the ability to scatter US waves back to the source. The signal produced by the MR depends on the intensity of the backscattered waves with respect to the signal produced by the surrounding medium. As the backscattered signal intensity is proportional to the scattering cross-section of the objects^23^, due to their small size MRs may cause poor scattering, resulting in weak US echoes. Most of the works reported in the state of the art have overcome the problem by visualizing MRs in controlled experimental conditions, where homogeneous and scarcely echogenic media (e.g., eye vitreous humor) were used to improve image contrast^24,25^. On the contrary, most biological tissues are highly heterogeneous and echogenic and produce high contrast imaging artifacts that hinder MR detection and visualization. In an in-vivo scenario, imaging artifacts can introduce large detection errors, produce dangerous localization instabilities, and compromise MRs tracking. To address this problem, US contrast enhancement can be pursued either with the inclusion of contrast agents^26^, such as microbubbles, or by exploiting the Doppler effect^15^. The first strategy suffers from the gas bubbles short half-life, whereas the latter looks more promising. In fact, moving objects produce acoustic phase lags in the backscattered waves, known as Doppler shifts, which are proportional to the objects’ displacement^27^. According to this principle, researchers have recently investigated the possibility of exploiting ultrasound color Doppler imaging to visualize microrobots in motion^28,29^. However, traditional color Doppler imaging is sensitive to all motions occurring in the imaging plane, hampering specific MR motions detection in dynamic and echogenic backgrounds (e.g., biological tissues subject to physiological motions). Exploiting the specific acoustic phase modulation produced by characteristic MR motions, e.g., vibrations, can help to address this limitation by enabling to isolate MR displacements from background motions, and thus enabling robust MR detection^30^. This strategy can provide a contrast-enhancing mechanism for improved MR tracking in echogenic and dynamic backgrounds, where traditional US imaging modalities fail. However, to make this contrast enhancement mechanism viable for MR tracking, it should be extended to the most common and widespread MR motion patterns, such as rolling which is particularly efficient for MR navigation in different body districts (e.g., vascular system, urinary system, or gastrointestinal tract^31–33^).

To fill the aforementioned gap in US-based MR imaging, in this work the authors combine magnetically activated rolling locomotion of a cylindrical MR with real-time US tracking by detecting specific MR motion patterns through acoustic phase analysis. To simulate MR tracking in a realistic therapeutic task, a flexible detection algorithm was also developed to detect the MR both during rolling locomotion (for target reaching) and during the idle state, i.e., when the MR is in the target position and target reaching has to be verified. The approach was validated in a lumen of a tissue-mimicking phantom, both in static and counter flow conditions. The proposed detection strategy allowed to perform continuous MR tracking even in the presence of high contrast imaging artifacts by robustly detecting its centroid position over time and deriving features such as size and rotation frequency.

## Results and discussion

The proposed US contrast enhancement strategy employed for imaging and tracking is based on the specific acoustic phase modulation produced by MR motions. As a result of the Doppler effect, moving objects scatter US waves which are shifted in phase with respect to the incident wave. If a wave is propagating with wavelength $\lambda$ and encounters a moving object, the acoustic phase shift $\partial \varphi$ in the backscattered echo is proportional to object displacement along the direction of wave propagation $\partial {u}_{y}$.

1 $$\partial \varphi =\frac{4\pi }{\lambda }\partial {u}_{y}.$$

The acoustic frequency $f$, given by the time derivative of the acoustic phase, is thus shifted proportionally to the object velocity ${v}_{y}$.

2 $$\frac{d(\partial \varphi )}{dt}=\partial f=\frac{4\pi }{\lambda }\partial {v}_{y}.$$

**Figure 1:**
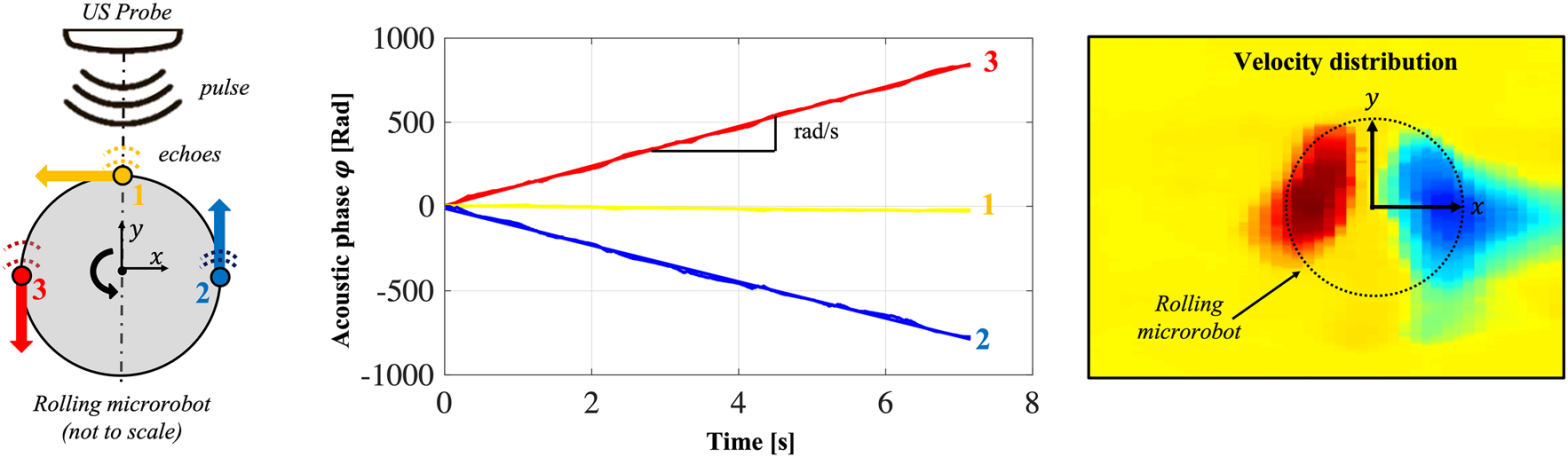
Acoustic phase shifts in the backscattered echoes induced by MR rotations. The dashed line represents the direction of wave propagation, namely the acoustic axis (y). Points in positions 2 and 3 continuously move respectively towards and out from the US probe, producing a continuous shift (with opposite sign) in the acoustic phase of received echoes. On the other hand, points in position 1 and all points along the acoustic axis y are not moving with respect to the probe. These rotating motion features produce a specific velocity distribution, which can be exploited for MR detection and visualization.

Therefore, acoustic phase signal $\varphi$ analysis provides information on object displacement and velocity along the acoustic axis (i.e., the direction of wave propagation), independently on the amplitude of the backscattered echoes. This phenomenon can improve the detectability of moving objects even when they produce weak echoes with respect to the background. To exploit this principle as a contrast enhancement mechanism, we analyzed the rolling motion features. A cylindrical MR rolling on a boundary will appear in the imaging plane of the US probe as a rotating circle (Fig. 1). Given the MR with radius $r$ and angular velocity $\omega$, the linear velocity ${v}_{y}$ of a generic point $\left(x,y\right)$ on the MR body, with respect to a reference system placed in the center of rotation is given by

3 $$ \begin{array}{*{20}l} v_{y} \left( {x,y} \right) = \omega { }\sqrt {x^{2} + y^{2} } {\,\text{ cos}}\left( {{\,\text{atan}}\left( \frac{y}{x} \right)} \right) \hfill \\ {\,\text{For }}\sqrt {x^{2} + y^{2} } \le r. \hfill \\ \end{array} $$

According to Eq. (3), ${v}_{y}$ is null when $x=0$ (e.g., point 1 and any point along the acoustic axis *y* in Fig. 1), and maximum for $\left|x\right|=r$ (points 2 and 3). In fact, the linear velocity of points along the *y* axis is orthogonal to the US wave propagation direction and does not produce any phase shift in the backscattered echoes (curve 1 in Fig. 1). On the other hand, the linear velocities of points with non-null x coordinate, i.e., all points outside the *y* axis, have a component parallel to the US wave propagation direction and produces a continuous phase drift in the backscattered echoes (curves 2 and 3). This characteristic velocity distribution can be exploited as a special signature to detect and visualize the rotating MR in the imaging plane, by properly processing the acoustic phase signal.

**Figure 2:**
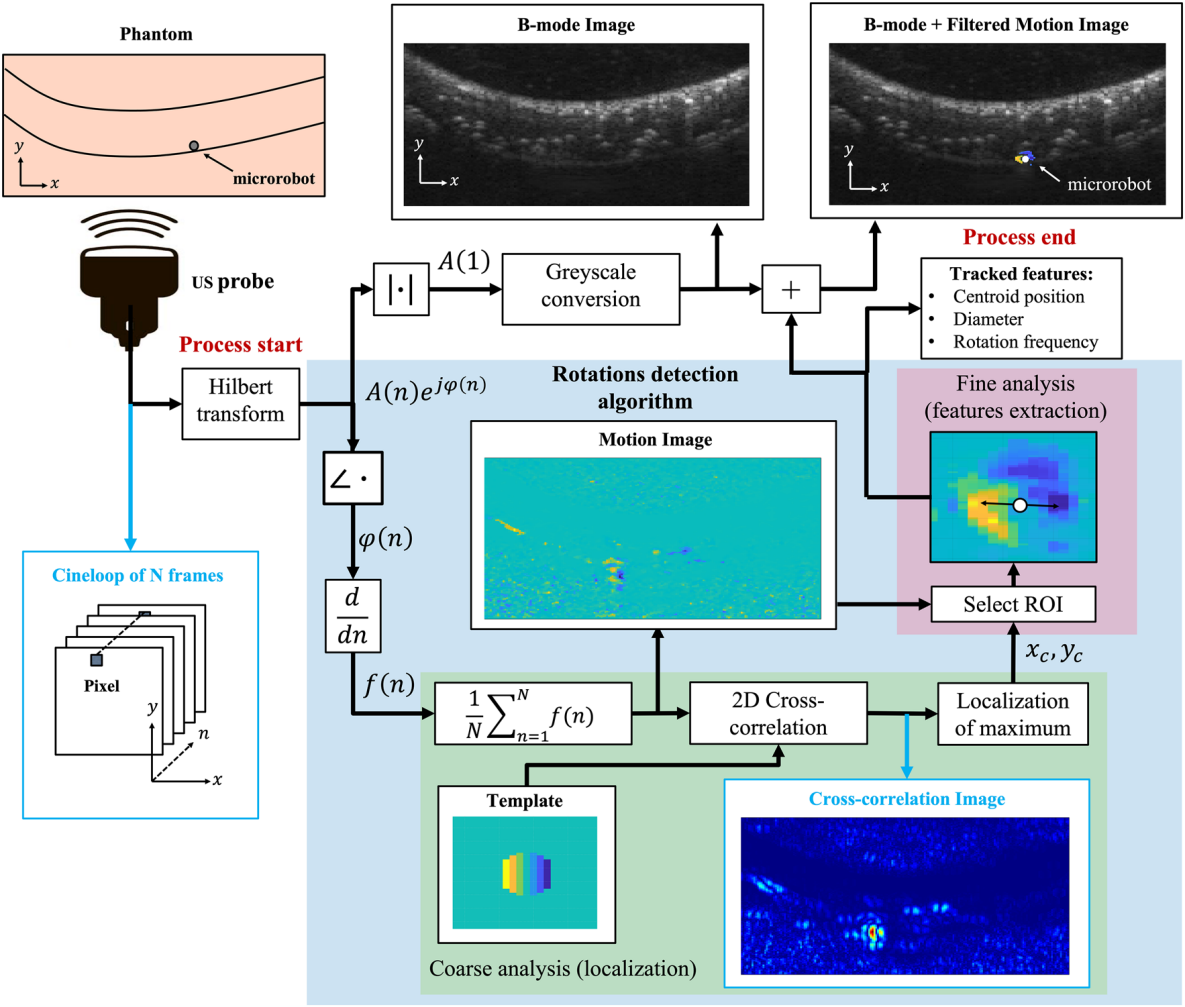
Acoustic phase processing for enhanced contrast US imaging and tracking of a rotating microrobot. Algorithm flow chart: Ultrasound RF data are acquired in the form of a cineloop from the probe. For each pixel in the cineloop, the analytic acoustic signal in the time dimension (n) is obtained through the Hilbert transform from the raw RF data. The instantaneous amplitude A is used for B-mode imaging, while the instantaneous phase ${\upvarphi }$ is analyzed for tracking the rotating MR (light blue box). Fast MR localization is achieved through a coarse phase analysis over the whole image plane (green box). Then a fine analysis is performed in a ROI defined around the centroid and features as MR diameter and rotating frequency are estimated by analyzing the velocity distribution (pink box). Finally, the selected ROI (Filtered Motion Image) is overlapped on a B-mode image for enhanced imaging and tracking. Illustrative outcomes of the process along the pipeline are displayed by the bright blue arrows in the flow chart.

Based on this principle, we developed an acoustic phase analysis algorithm for dynamic tracking of rotating MRs. For the benefit of time efficiency, which is a requirement for real-time imaging, the algorithm works at two different levels: (i) a coarse analysis is performed over the whole imaging plane, to allow for fast MR localization by identifying the MR centroid position; (ii) a finer and more computationally demanding analysis is then performed only on a reduced region of interest (ROI), defined in the identified centroid neighborhood, so as to derive MR features. The coarse analysis (localization) starts with raw RF data acquisition from the US transducer (Fig. 2). The RF data associated with $N$ consecutive frames constitute a 3D matrix called cineloop. The first two dimensions of the cineloop, $(x)$ and $(y)$, represent the lateral and axial dimensions for each frame, respectively. The third dimension $(n)$ represents the acquired frames index in time. The analytic acoustic signal ${E}^{*}$ is obtained from the RF data cineloop through the Hilbert transform. For every pixel $(x,y)$ in the cineloop, ${E}^{*}$ along the time dimension $n$ is expressed as:

4 $${E}^{*}\left(n\right)=A\left(n\right)\cdot {\,\text{e}}^{j\varphi \left(n\right)}.$$

$A(n)$ is the instantaneous amplitude, which is converted into greyscale levels to obtain the pixel intensity for B-mode images^23^. The instantaneous phase $\varphi (n)$, which carries information on objects’ displacements, is time derived to obtain the instantaneous frequency $f(n)$. The average of $f(n)$ over the $N$ acquired frames provides a 2D image representing the mean velocity for each pixel, which is defined as the Motion Image (MI) (Fig. 2, in the center). Rotating objects in the MI produce the velocity distribution described in Eq. (3). Therefore, to detect a rotating MR, the MI is cross-correlated with a velocity template, defined by Eq. (3). The template considers unitary rotation frequency $\omega$ since the maximum cross-correlation is expected in the MR centroid regardless of its rotation frequency. This condition stands true as long as no other circular object with comparable size to the MR is continuously rotating in the imaging plane. Once identified the MR centroid, a ROI centered in its position is defined, and the fine analysis is started. A Filtered Motion Image (FMI) is obtained from the ROI and is analyzed to estimate MR features. The MR diameter is estimated as the distance between the pixels in the FMI featuring the maximum and minimum intensity (velocity), respectively (as in points 2 and 3 of Fig. 1). The MR rotation frequency is given by the maximum pixel intensity in the FMI, which corresponds to the tangential velocity on the MR surface (as again in points 2 or 3 of Fig. 1), divided by the radius (half the estimated diameter). Finally, the FMI is overlapped with the B-mode image to perform enhanced imaging and tracking. This allows integrating morphological and anatomical information on the environment provided by B-mode with specific MR information provided by FMIs.

**Figure 3:**
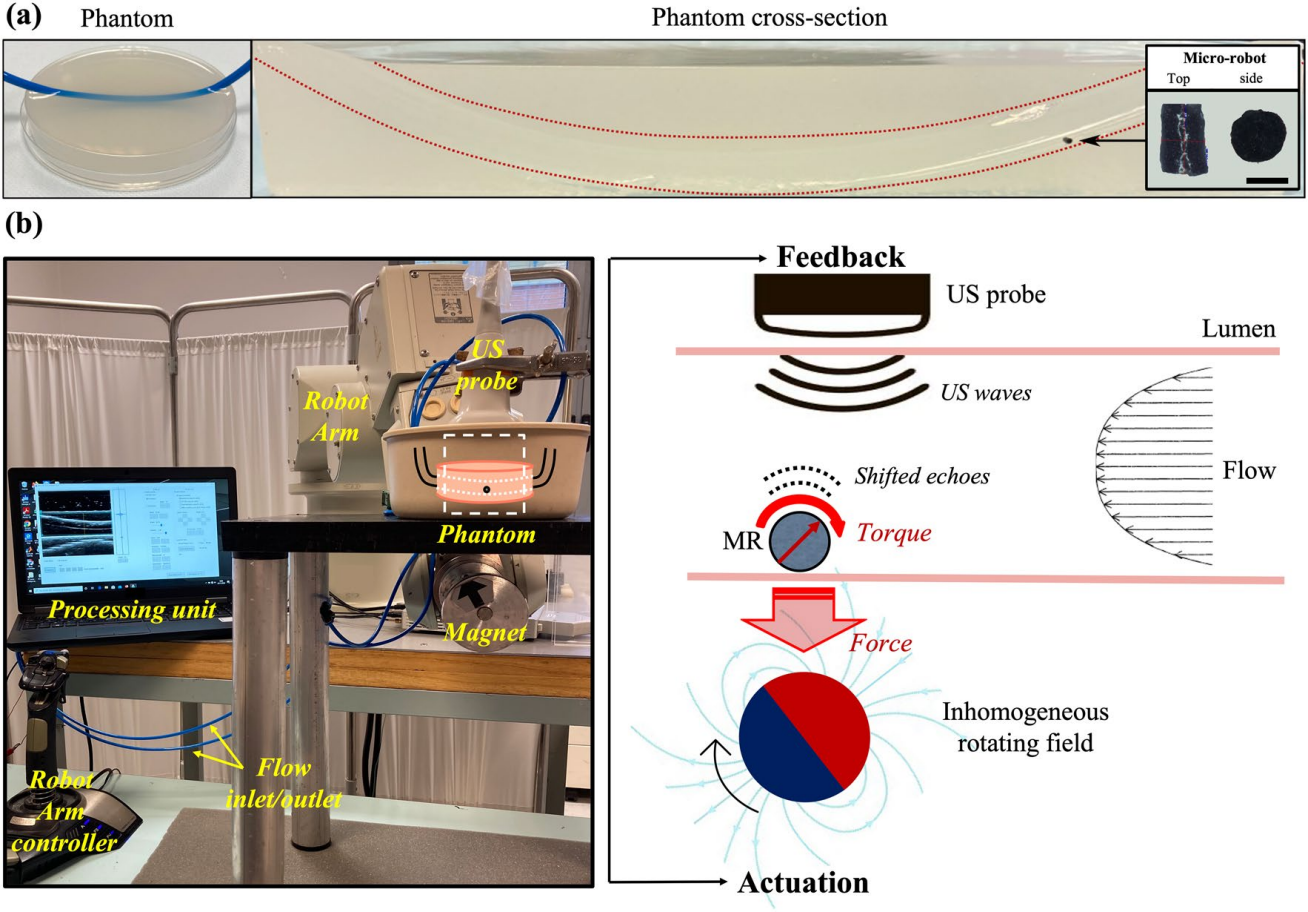
US-guided magnetic navigation platform. (**a**) The cylindrical magnetic microrobot is positioned inside the lumen of a tissue-mimicking phantom. The scale bar is 500 ${\upmu m}$. (**b**) The MR is actuated by means of external magnetic fields produced by a permanent magnet mounted to the tip of a robot arm. The pulling force generated by the magnetic field gradients was exploited to migrate the MR at the lumen boundary and grant contact to its surface, allowing for magnetic torque-activated rolling locomotion. The flow in the lumen is produced by a fluidic circuit supplied by a micropump. A standard US probe is used for continuous imaging of the phantom, and a Desktop computer is used for RF data processing, tracking, and visualization.

To validate the proposed tracking strategy in an in-vivo like environment, we navigated a cylindrical magnetic MR (550 μm diameter, 990 μm length) through a tissue-mimicking phantom with a lumen (3 mm diameter) (Fig. 3a). The magnetic MR was placed in the lumen and actuated through external inhomogeneous magnetic fields, generated by a cylindrical permanent magnet rotated through a robotic arm. The pulling force generated by the magnetic field gradients was exploited to migrate the MR at the lumen boundary and grant adherence to its surface, a precondition for rolling locomotion (Fig. 3b). The rotation of the permanent magnet around its axis produced a magnetic torque aligning the magnetic moment of the MR with the external field, thus activating rolling locomotion. To demonstrate the robustness of the tracking approach in echogenic and moving environment, a flow of blood-mimicking fluid was generated inside the lumen ($3 \,\text{mL} \,{\,\text{s}}^{-1}$) in the opposite direction of MR locomotion. For tracking the MR, the phantom was imaged with standard clinical US equipment while the raw Radio-Frequency (RF) data were processed by custom algorithms implemented on a desktop computer.

**Figure 4:**
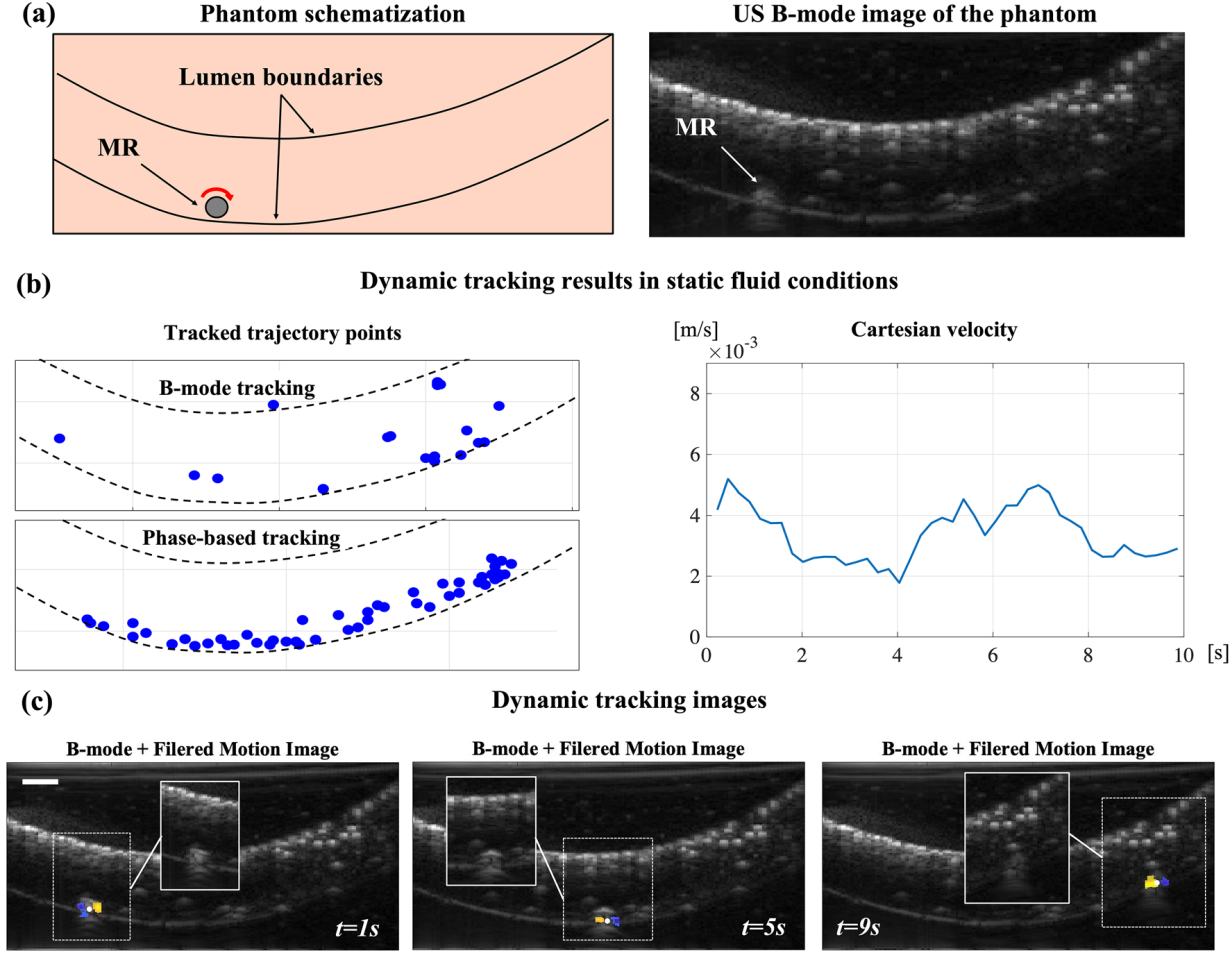
Dynamic MR tracking in echogenic background. (**a**) Schematization of phantom for tracking experiments and corresponding B-mode image. (**b**) Results of trajectory tracking in static fluid conditions. The tracked trajectory points graph show how B-mode tracking fails due to static objects with higher contrast than the MR (e.g., lumen boundaries). The phase-based approach succeeds in tracking the MR along the entire trajectory. The cartesian velocity plot shows an almost constant trend, suggesting that the MR performs a nearly pure rolling motion. (**c**) Dynamic tracking images for different time instants during the experiment. The filtered motion images show MR contrast enhancement enabling robust detection even when the MR is barely visible in standard B-mode images (shown in image insets). The scale bar is 1 mm.

First, we validated the performances of the algorithm in estimating the rolling MR features for variable diameters and rotation frequencies. The algorithm resulted accurate with a maximum estimation error of 3% and 13%, for diameter and frequency, respectively (see Sect. B of Supplementary Information and Supplementary Fig. S2). Then, we conducted navigation experiments to demonstrate the method potential for dynamic tracking of MR trajectories in the presence of high contrast imaging artifacts. To this aim, we rolled the MR on the boundary of the fluid-filled lumen of the tissue-mimicking phantom under continuous US imaging (Fig. 4a), first in static fluid conditions and later with an induced flow, opposing the rolling direction. In all the experiments, the MR was actuated with a magnetic field rotating at the constant frequency of 1.5 Hz and monitored over a 10 s acquisition time with a tracking frame rate of $3\,\text{fps}$. During navigation, we compared the performances of the proposed tracking algorithm with those of a B-mode algorithm based on image differentiation, particularly suitable for tracking rotating objects^22^.

In the static fluid experiments, by analyzing the tracked trajectory points (Fig. 4b), we observed that the B-mode tracking algorithm intermittently failed in localizing the MR. This was caused by static echogenic background elements, e.g., the lumen boundaries, which showed considerably higher contrast than the MR. In fact, most of the tracked points were concentrated in few locations along the lumen boundary, corresponding to the highest contrast pixels in the image. On the other hand, the phase analysis-based algorithm allowed to robustly track the MR positions along the entire trajectory. The stable tracking of the MR centroid positions allowed to estimate the cartesian velocity by time derivation. The velocity profile showed minimal fluctuations around $2.6 \,\text{mm}\,{ \,\text{s}}^{-1}$, which corresponds to the expected linear velocity of a cylinder with a diameter of 550 μm, rolling with an angular frequency of 1.5 Hz. This result confirmed that, in static fluid conditions, the MR performed an almost pure rolling locomotion with very little slip (see Supplementary Video 1). By overlapping the FMIs acquired during different time instants of the MR trajectory with a static B-mode background image (Fig. 4c), we could verify two aspects: (i) despite the low contrast of the MR relatively to the environment (image insets in Fig. 4c), the specific phase signature produced by its motion allowed for enhanced visualization and detection inside the lumen; (ii) throughout the entire trajectory, the MR centroid was precisely localized. In particular, assuming pure rolling and rigid bodies, the average centroid tracking error was estimated to be 135 μm (about 0.25 body-lengths), defined according to the distance from the lumen wall.

**Figure 5:**
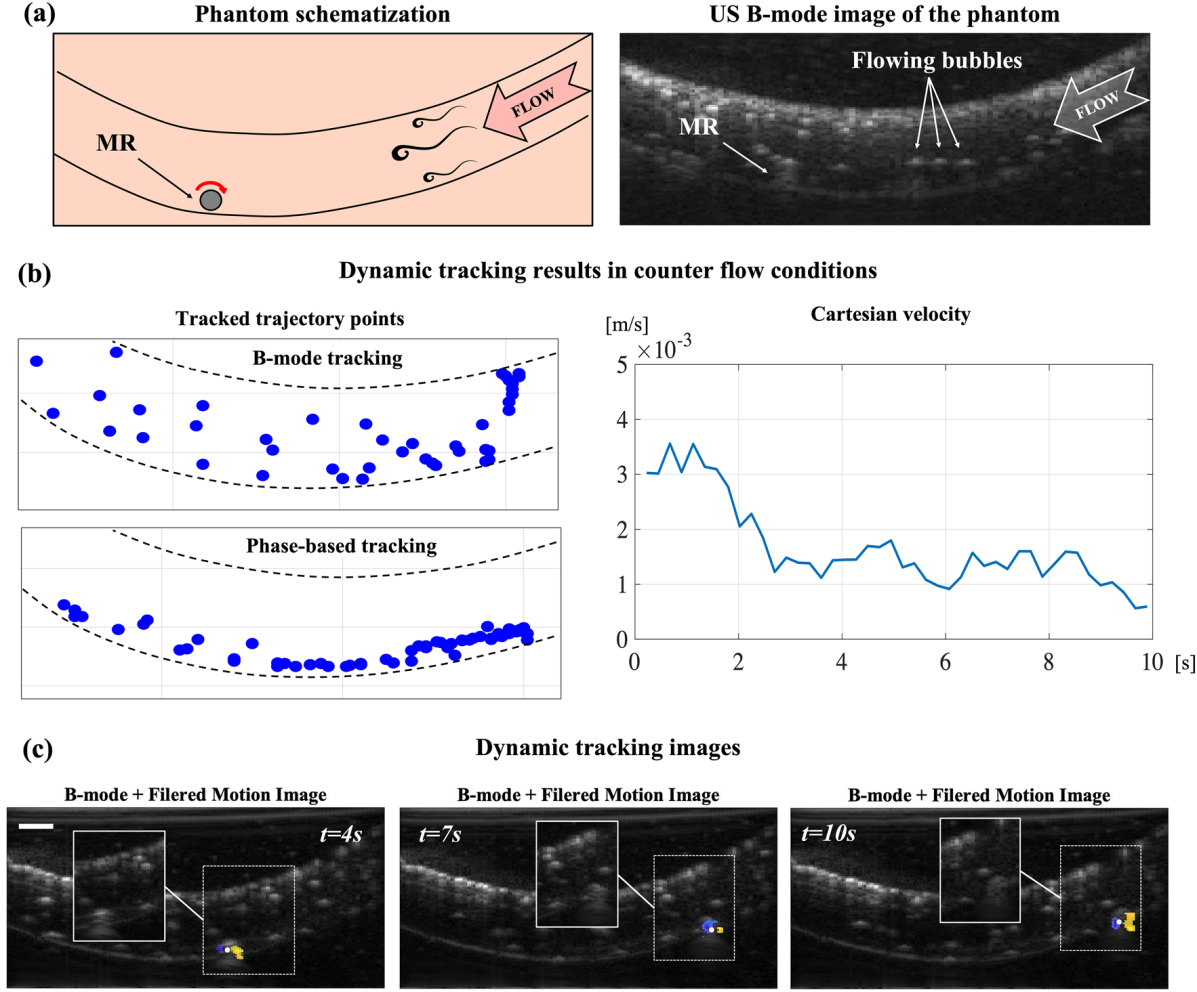
Dynamic MR tracking in echogenic counter fluid flow. (**a**) Schematization of phantom for tracking experiments and corresponding B-mode image. (**b**) Results of trajectory tracking in the presence of an induced flow. The tracked trajectory points graph shows how the B-mode tracker is disturbed by distributed high contrast particles in the flow (bubbles), while the phase-based tracker can follow the MR robustly. The trend in the Cartesian velocity plot shows how the presence of an opposite flow introduces slip in the rolling motion, reducing the MR velocity until it eventually approaches zero when the MR runs across the inclined section of the lumen (t = 10 s). (**c**) Dynamic tracking images for different time instants during the experiment. The filtered motion images show MR contrast enhancement enabling robust detection even when the MR is barely visible in standard B-mode images (shown in image insets). The scale bar is 1 mm.

The navigation experiments were then repeated when applying a $3\,\text{mL} \,{\,\text{s}}^{-1}$ flow opposing the MR locomotion direction. The pump induced the creation of microbubbles appearing in the B-mode images as echogenic moving objects (Fig. 5a). In this case, by analyzing the tracked trajectory points (Fig. 5b) we observed that—with the B-mode algorithm—they were almost uniformly distributed around the lumen region. This result suggests that in the presence of fluid flow the B-mode tracker was primarily disturbed by the flow generated bubbles, rather than by the lumen boundaries. On the other hand, even in presence of these echogenic moving objects, the proposed phase-based algorithm proved efficient in robustly tracking the MR along the entire trajectory. The average position tracking error was in line with previous experiments (less than 0.25 body-lengths). With the addition of an opposing flow, as the MR approached the inclined part of the lumen on the right side of the image (t = 3 s in Fig. 5b), the cartesian velocity started to decrease, eventually reaching zero. This behavior reflected that the rolling motion, in this case, featured a slip between the MR and the boundary, which hampered the MR progression (see Supplementary Video 1). The FMIs overlapped on the B-mode background for different time instants (Fig. 5c) allowed once again to verify that the MR contrast enhancement of phase analysis is not disturbed by the presence of flowing bubbles, and the MR can be isolated from the background even in the presence of more echogenic moving objects.

**Figure 6:**
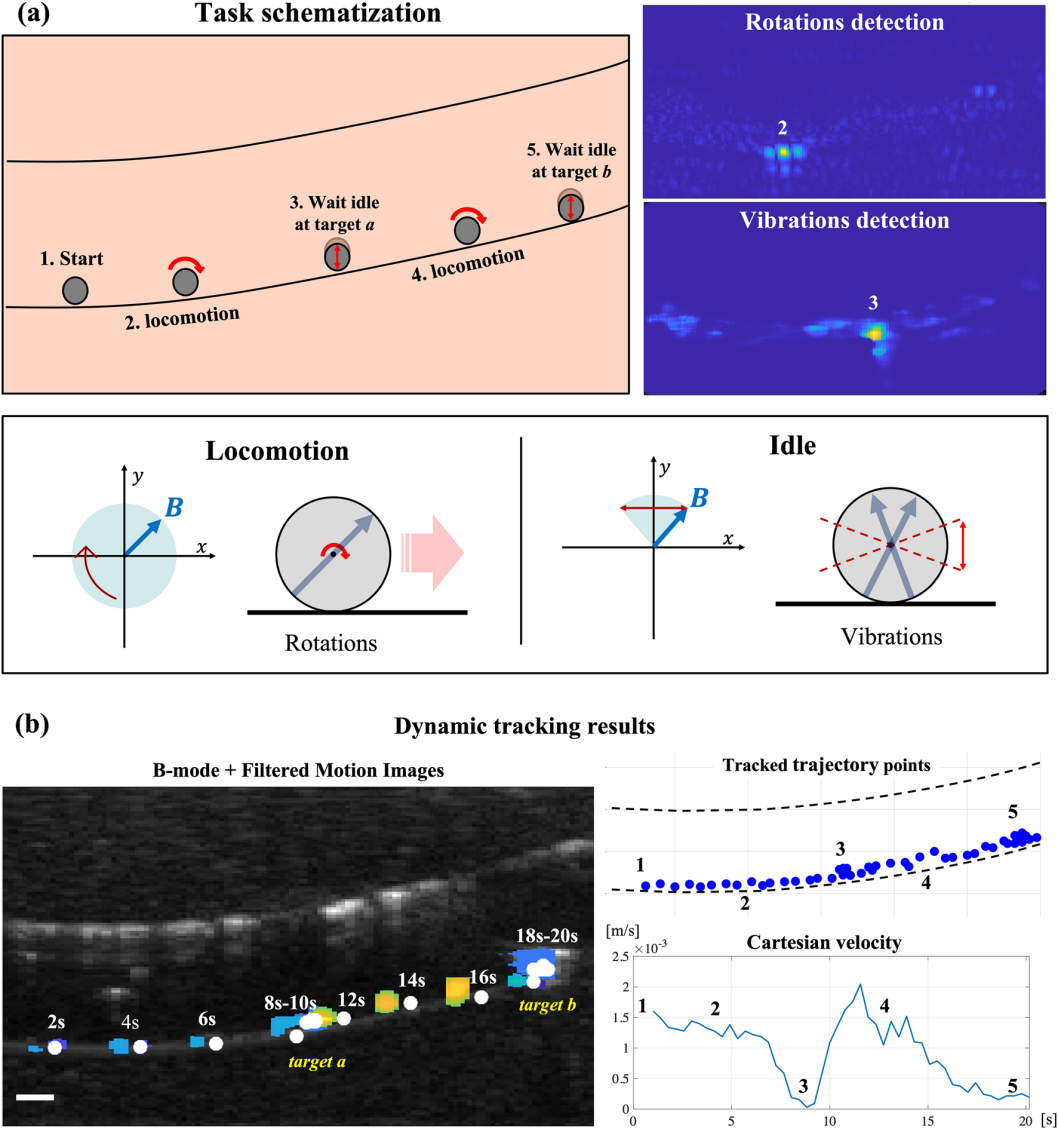
Phase-based tracking in a multi-modal navigation task. (**a**) In the defined navigation task, the MR can assume two different states: rolling and idle. A rotating magnetic field activates rolling locomotion state while, in the idle state, micro-vibrations are induced by harmonic oscillations of the magnetic field. Two different phase analysis strategies are performed according to the MR status, namely rotations detection and vibrations detection. (**b**) Results of the multi-modal navigation tracking experiments. The dual tracking approach allowed for robust continuous tracking of the MR during the entire task. The estimated cartesian velocity faithfully reports the expected MR behavior during the different navigation task phases (1–5). The scale bar is 1 mm.

Lastly, we aimed at demonstrating that the proposed phase-based tracking approach can be adopted to continuously track the MR, not only during rolling locomotion but also when it is idle in place. To this purpose, we conducted additional experiments in a multi-modal MR navigation task (Fig. 6, Supplementary Video 1) in which the microrobot could assume two states: the rolling state (i.e., employed to reach the target location) and the idle state (i.e., once reached the target). During the rolling state, the MR was actuated by a rotating magnetic field and detected through its characteristic rotations, with the algorithm proposed in this paper (see Fig. 2); during the idle state, the magnet was rotated to produce a harmonic motion over a circular sector so as to induce in-place micro-vibrations of the MR, at a frequency of $5\,\text{Hz}$ (Fig. 6a). These vibrations allowed to track the MR through frequency analysis of the acoustic phase signal, by using a vibrations detection algorithm previously reported by the authors^30^. When passing from one state to the other, the robot arm controller (which actuated the permanent magnet) communicated the MR status to the tracker, which could switch detection modality accordingly.

For this experimental validation, we designed a simple task: the MR is navigated from the start point (1) to the target point *a* by rolling motion (2). When the target *a* is reached (3), the MR waits idly in place (vibration state) for $2\,\text{s}$. After this stage, in which the reaching of the target location can be verified (i.e., in a real in vivo environment by identifying potential anatomical markers), the MR is actuated again in rolling state (4) towards target *b*. Once target point *b* is reached, the MR waits idly again for $2\,\text{s}$ and finally completes the task (5). The FMIs overlapped with B-mode show the MR positions in the lumen at different time instants, demonstrating successful continuous tracking over the entire trajectory (Fig. 6b). The estimated position tracking error was in line with previous experiments (less than 0.25 body-lengths). The time evolution of cartesian velocity faithfully reflected the MR behavior during the different navigation task phases: velocity was almost constant during rolling through task phases 2 and 4. During task phases 3 and 5, the velocity was close to zero as the MR was waiting in place idle, and the tracked trajectory points concentrated in the target locations *a* and *b*.

## Conclusion

In conclusion, we proposed a simple yet powerful strategy to improve MR visualization with US in echogenic media, such as biological tissues. Particularly, we exploited the specific acoustic phase modulation produced by characteristic MR motions as a contrast-enhancing mechanism for robust real-time imaging and tracking in high-contrast backgrounds, where standard modalities fail. Position tracking performances were validated through navigation experiments conducted in a tissue-mimicking phantom, both in static fluid conditions and then with an opposing flow. Furthermore, we demonstrated the applicability of the phase analysis approach not only when the MR is locomoting, but also when it is idle in place, provided it is continuously producing phase feedback (e.g., rotations or vibrations). In all experiments, the proposed approach allowed direct MR visualization and robust position estimation over the entire trajectories, with an average tracking error of about 0.25 body-lengths, outperforming B-mode, which provided poor MR visualization and produced substantial tracking errors (up to 40 body-lengths) due to background disturbances. Furthermore, we could estimate MR diameter and rotation frequency with a maximum estimation error of 3% and 13%, respectively. Although the MR presented in this study featured fixed size and constant rotation frequency, real-time estimation of these features can be a powerful tool when applied to shape morphing MR^34^ or nanoparticles swarms^35^.

The temporal resolution of the proposed methodology is related to the MR motion dynamics. In fact, the tracking frame rate depends on the observation time required for MR localization. This time interval is shorter if the target MR motions exploited for detection have fast dynamics (e.g., high rotation or vibration frequency), but may be larger if they are slow. A low tracking frame rate could also limit the maximum trackable MR translational velocity. In fact, signal distortion could happen if the MR translates too much during a long localization observation time, potentially causing tracking failure. For these reasons, to enable high tracking frame rates, higher MR motion dynamics are preferable, provided their time constants are within the Nyquist frequency limits imposed by the phase signal sampling period. On the other hand, the spatial resolution is limited by the pixel size in the US frames, which is generally defined by the probe design parameters. For example, the B-mode pixel size is around 150 µm × 150 µm for commercially available linear High-Definition probes. However, phase analysis allows for improved resolution since the axial pixel size in FMIs is reduced by a factor of four (down to 37 µm)^30^. Furthermore, in contrast to the acoustic intensity, the acoustic phase is not affected by tissue attenuation, and we found acoustic phase shifts to be proportional to MR motions, independently of the distance from the transducer. These principles could allow, in theory, for high-resolution imaging at considerable penetration depths.

Overall, the results reported in this study validated acoustic phase analysis as a successful approach for visualizing and tracking MRs in echogenic backgrounds and during different task states, offering new insights into smart combinations of actuation and imaging at the microscale. In fact, the proposed contrast-enhancing mechanism is not only limited to detecting rotating or vibrating motion patterns. Instead, it is more generally based on detecting any displacement which has a projection along the direction of wave propagation. This condition can be found in several MRs actuation approaches, including different navigation and function activation strategies^1,36,37^. In this context, future developments of this work shall investigate the impact of different MR geometries and physical properties on their interaction with US waves. In conclusion, the perspectives stemming from this study unveil the possibility of exploiting acoustic phase analysis as a robust feedback strategy for closed-loop control of biomedical MRs in-vivo.

## Methods

### Tissue-mimicking phantom

To validate the proposed tracking strategy in an in-vivo like environment, we wanted to mimic the discontinuous acoustic properties of heterogeneous tissues. In fact, acoustic impedance discontinuities produce US waves reflection and act as high contrast elements which hinder MR visualization with contrast-based imaging methods (e.g., B-mode). In order to mimic these conditions, we fabricated a rather simple phantom, made of tissue-mimicking hydrogel doped with scatter-enhancing agent and provided with a fluid-filled luminal cavity. Such a phantom features acoustic impedance discontinuities at the scatter-enhancing agent doped regions and at the interfaces between the hydrogel and the lumen cavity, which produce high contrast elements and allow to validate the proposed technique in a highly echogenic environment. The tissue-mimicking phantom was made from agarose gel and soy milk used as a scatter-enhancing agent. Agarose powder (Sigma-Aldrich) was dissolved in deionized and degassed water (dd-H2O)—soy milk (5% v/v) solution and kept at $90\,^\circ \text{C}$ for $1 \,\text{h}$ under continuous stirring. Selecting the proper agarose concentration (2% v/v) produces mechanical and acoustic properties that mimic human tissues^38^. Physical reticulation occurred at room temperature in the target mold (standard Petri dish). A 3 mm diameter rubber tube was embedded in the phantom before reticulation. After reticulation the tube was removed to generate the desired luminal cavity in the phantom.

### Blood-mimicking fluid

A fluid that mimics the viscosity and acoustic properties of blood was obtained from an aqueous glycerol solution (60% v/v)^39^.

### Rolling microrobot

To perform controlled rolling over the lumen internal surface through external magnetic fields, we required a sub-millimeter cylindrical MR with remanent magnetization along the radial direction. For this purpose, we employed extrusion-based printing of a UV curable magnetic ink. For more details regarding MR fabrication and design choices, the reader may refer to Section A of the Supplementary Information and to the Supplementary Fig. S1. To achieve uniform radial magnetization, the printed cylindrical string was magnetized radially by an impulse magnetizer with a peak field intensity of $1.8 \,\text{T}$ (T-Series, Magnet-Physik Dr. Steingroever GmbH, Germany). The final length of the cylindrical MR was defined by cutting the magnetized string into smaller segments with required aspect ratio (2:1) by using a scalpel under microscope guidance. The resulting MR length and diameter were measured respectively around $990 \upmu \,\text{m}$ and $550 \upmu \,\text{m}$, using a digital microscope (KH-7700, Hirox Co., Ltd, Japan), and the remanent magnetization was measured $27.5 \pm 1.7 \,\text{emu}\,{\text{g}}^{-1}$, using a Vibrating Sample Magnetometer (Model10 VSM, MicroSense, USA).

### Experimental platform for MR navigation

To validate the proposed tracking strategy, we built an experimental platform to navigate the magnetic MR through the lumen of the phantom. The magnetic MR was placed in the lumen and actuated through external inhomogeneous magnetic fields, generated by a cylindrical permanent magnet ($6 {\,\text{cm}}$ in diameter, $7 {\,\text{cm}}$ in height, NdFeB, diametral magnetization, grade N35) positioned $10 \,\text{cm}$ far away from the phantom and rotated through a robotic arm (Melfa RV-3S, Mitsubishi, Japan). The pulling force generated by the magnetic field gradients was exploited to migrate the MR at the lumen boundary and grant adherence to its surface, a precondition for rolling locomotion. The rotation of the permanent magnet around its axis produced a magnetic torque aligning the magnetic moment of the MR with the external field, thus activating rolling locomotion. A micropump (M100S, TCS micropumps) injected the blood-mimicking fluid through the lumen with a flow speed of $3 {\,\text{mL}}\,{\text{s}}^{-1}$. The flow allowed to demonstrate the robustness of the tracking approach in heterogeneous and dynamic backgrounds. For tracking the MR, the phantom was imaged with a standard linear US probe (L15-7H40, Telemed, Lithuania). The raw RF data were acquired by an open architecture digital beamformer (ArtUS, Telemed, Lithuania) and processed by custom software implemented on a desktop computer (XPS, Dell, France).

## Supplementary Information

Supplementary Information.

Supplementary Video 1.

## Data Availability

The data that support the findings of this study are available from the authors upon reasonable request.
