# Supplementary material for: Dynamic tracking of a magnetic micro-roller using ultrasound phase analysis
Source: Sci Rep. 2021 Dec 1;11:23239. doi: 10.1038/s41598-021-02553-z (PMC8636564; doi:10.1038/s41598-021-02553-z)
Supplement: Supplementary file 1 — Supplementary Information. [file 41598_2021_2553_MOESM1_ESM.pdf]

Dynamic tracking of a magnetic micro-roller using ultrasound phase analysis

Stefano Pane[1,2,*], Veronica Iacovacci[1,2,3,*], Mohammad Hasan Dad Ansari[1,2,4], and Arianna Menciassi[1,2]

[1]The BioRobotics Institute, Scuola Superiore Sant'Anna, Pisa, 56025, Italy
[2]Department of Excellence in Robotics and AI, Scuola Superiore Sant'Anna, Pisa, 56025, Italy
[3]Department of Mechanical and Automation Engineering, The Chinese University of Hong Kong, Shatin, Hong Kong, SAR
[4]Robot-Assisted Surgery Group, Department of Mechanical Engineering, KU Leuven, Leuven, 3001, Belgium
[*]stefano.pane@santannapisa.it, veronica.iacovacci@santannapisa.it

Supplementary Information

A. Microrobot fabrication

In this work we targeted the case study of a microrobot rolling over a boundary surface under the effect of external driving fields. The MR was designed and fabricated in the attempt to optimize both the rolling performance and its tracking. Selecting the shape, we opted for a cylindrical body to maximize the adhesion surface, thus optimizing rolling performances. The size of the cylindrical body was selected to be in the sub-millimeter range, namely 550μm in diameter and 990μm in length, so as to fit the target application of navigating lumina with millimetric diameter (3mm). To fulfil these design criteria, we fabricated a cylindrical string adopting an extrusion-based printing technique (Figure S1). The extrudable ink was prepared using a UV curable elastomer and NdFeB particles. The NdFeB powder provided UV curable elastomer with magnetic properties and also enhanced scattering properties, slightly increasing its otherwise low echogenicity.
UV enables fast curing which can help develop a high throughput fabrication process, while NdFeB was selected due to its strong magnetic properties. The UV curable elastomer (UV Electro 225-1, Momentive, New York, USA) and a UV catalyst (UV LSR catalyst, Momentive, USA) were mixed with NdFeB powder (MQFP-15-7, Magnequench, Singapore) in a 1:0.1:1 weight ratio in a speed mixer (ARE 250, Thinky, Japan), in a closed container. After mixing the individual components, the resulting mixture was transferred to a syringe and permanently magnetized in an Impulse Magnetizer with a peak field intensity of 1.8T (T-Series, Magnet-Physik Dr. Steingroever GmbH, Germany). This magnetization step, combined with the high viscosity (70 Pa · s) of the UV elastomer, helps retain the cylindrical shape during extrusion. A syringe tip (0.58 mm) was then attached to the syringe and the ink was slowly extruded using a bioplotter (Omega, 3Dynamic Systems, UK). The diameter of the syringe tip defines the final MR diameter. The printer bed was positioned 3cm below the syringe tip to allow the extruded ink to cure into cylindrical strings using focused UV light (385 nm). Curing the elastomer right after it is extruded from the syringe tip and before it is collected on the printer bed further helps retain the final MR's cylindrical shape. As a result of the shear alignment of the magnetic particles, the cylindrical string had non-uniform magnetization biased towards the axial direction. To achieve uniform radial magnetization, the cylindrical string was then re-magnetized radially by the impulse magnetizer. The final length of the MRs was defined by cutting the magnetized cylindrical string into smaller segments with required aspect ratios by using a scalpel under microscope guidance. The resulting MR length and diameter were measured respectively around 990 μm and 550 μm, using a digital microscope (KH-7700, Hirox Co., Ltd, Japan), and the

remanent magnetization was measured using a Vibrating Sample Magnetometer (Model10 VSM, MicroSense, USA). The MR remanent magnetization was 27.5 ± 1.7 emu/g.

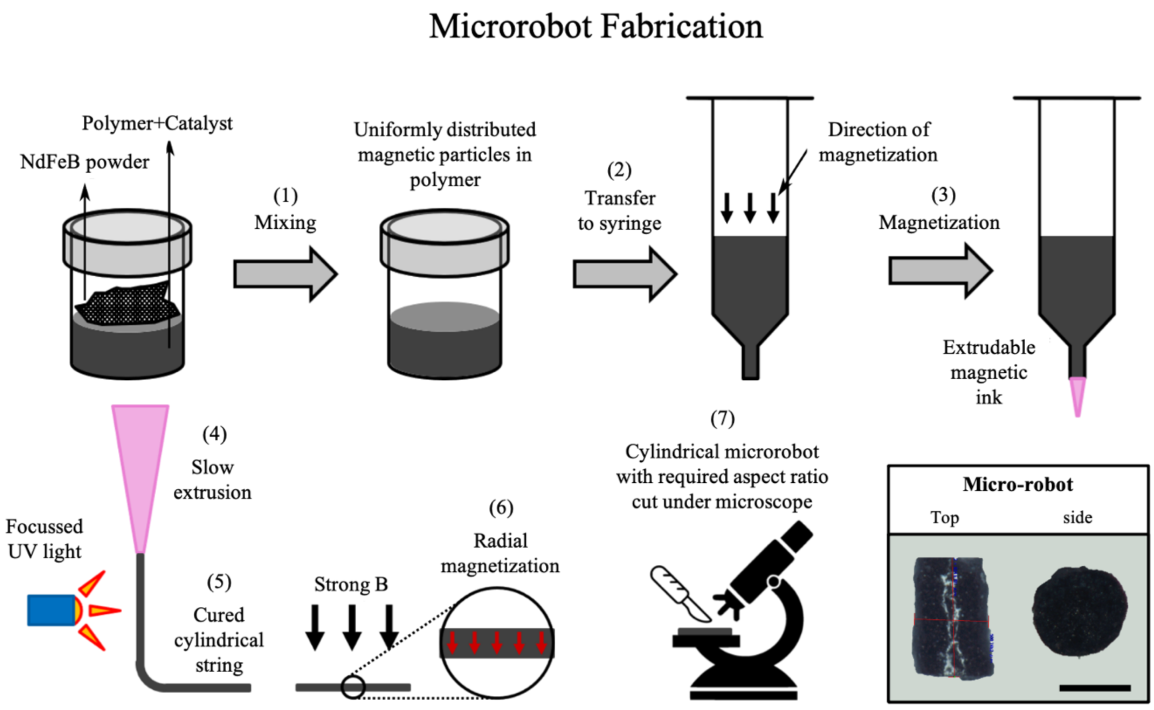

Figure S1. Microrobot fabrication: UV curable polymer along with a UV catalyst is mixed with magnetic powder (1), and the resulting mixture is transferred to a syringe (2) and permanently magnetized (3), thus resulting in an extrudable magnetic ink. This ink is extruded through a bioplotter (4) and cured, forming continuous cylindrical strings (5), which are then re-magnetized radially (6). Segments of required lengths are cut under a microscope (7) to produce our microrobots, as shown in the images captured under a digital microscope. The scale bar is 500 µm.

B. Identification of microrobot's features from acoustic phase analysis

Before navigation experiments, we conducted a set of preliminary experiments to validate the ability of the algorithm presented in the main text to localize MR centroid and derive its diameter and rotation frequency. In these experiments, the aim was to observe the US phase signals related to simply rotating objects without any translation due to rolling locomotion. To this aim, we generated an in-place rotating motion of the MR by embedding it within a full agar phantom. In the presence of a rotating external magnetic field, the slip conditions at the interface between the MR and the surrounding agar allowed for rotation in place without locomotion (Figure S2). We employed MRs of different diameters (1mm, 2mm) and actuated them with different magnet rotation frequencies (0.2Hz, 0.4Hz, 0.6Hz) to obtain a uniform and constant MR rotating motion. For each MR size, we collected a total amount of 30 diameter measurements and 30 rotation frequency measurements, 10 for each input frequency. The pixel intensity distributions in the motion images showed strong similarity with the velocity distribution of eq. (3), with the presence of the two symmetric lobes for positive and negative velocities respectively (Figure S2). The MR centroid position was robustly localized in the center of symmetry of such distribution. The diameter measurements for the 1mm

and the 2mm MR were distributed around $0.97 \pm 0.03\ mm$ and $2.1 \pm 0.02\ mm$, respectively. The maximum relative error in the diameter estimation was 3%. Additionally, the rotation frequency measurements for both MR sizes showed very good agreement with real values, featuring an average relative error of 13%. A higher error in this measurement can be justified by unmodelled interactions between the MR and the surrounding agar phantom (e.g., frictions). Overall, the reported results validated the proposed strategy for estimating features.

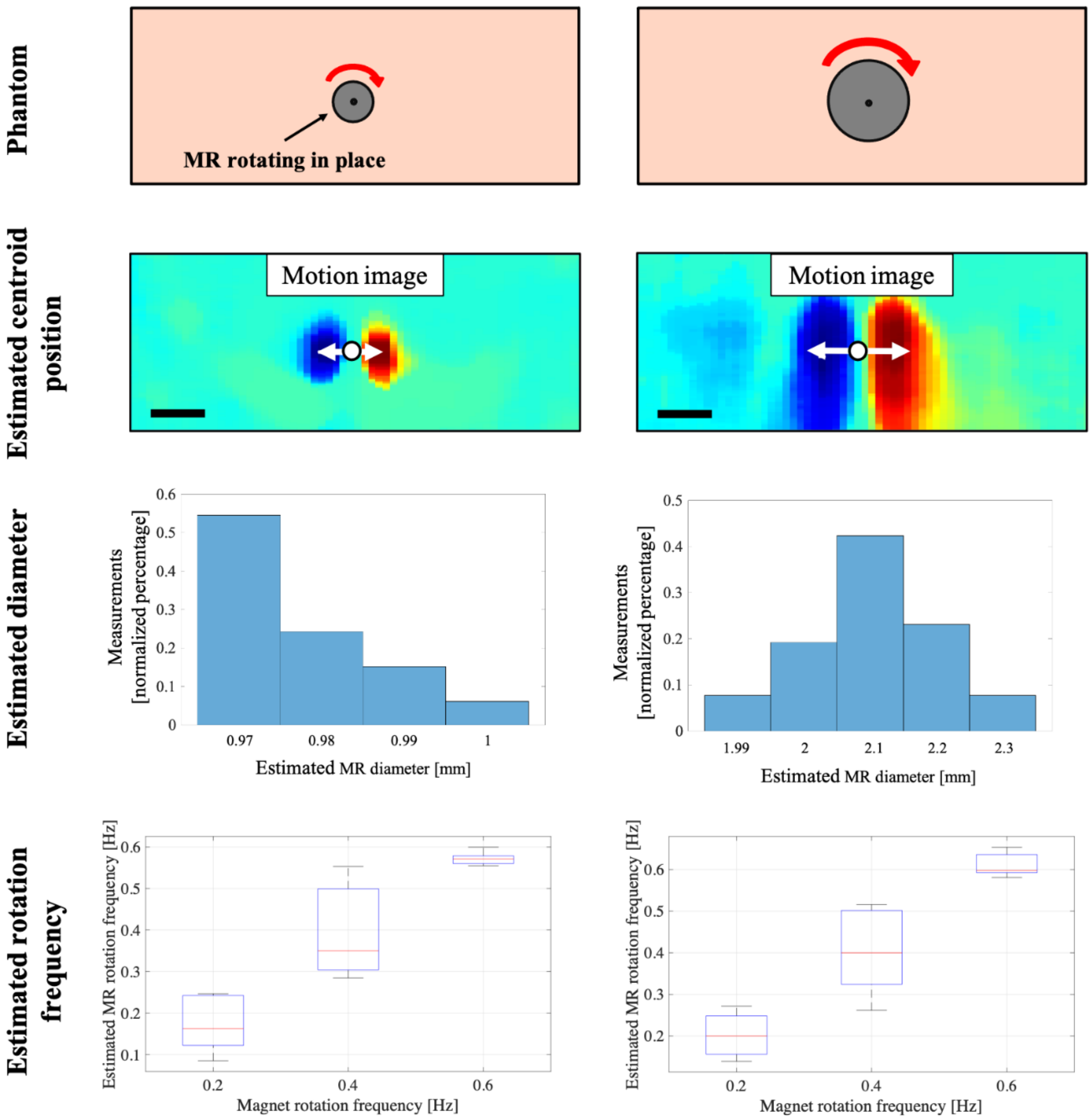

Figure S2. Analysis of the acoustic phase signals produced by rotating objects. The motion images acquired from MR of different sizes (1mm, 2mm) rotating at different frequencies (0.2Hz, 0.4Hz, 0.6Hz) are reported, together with the corresponding features estimated with the proposed algorithm. The pixel intensity distributions in the motion images are in full agreement with the theoretical models, and the centroid is robustly estimated in the velocity distribution center of symmetry. For both MR sizes, the diameter is estimated with a relative error below 3%, and the measured rotation frequencies reflect the respective magnet rotation frequency with an average relative error of 13%. The scale bar is 1mm.
